# INTRAPERITONEAL CHEMOTHERAPY FOR GASTRIC CANCER WITH PERITONEAL CARCINOMATOSIS: STUDY PROTOCOL OF A PHASE II TRIAL

**DOI:** 10.1590/0102-672020230026e1744

**Published:** 2023-07-17

**Authors:** Marcus Fernando Kodama Pertille Ramos, Marina Alessandra Pereira, Amir Zeide Charruf, Carolina Ribeiro Victor, João Vitor Antunes Marques Gregorio, Luciana Bastos Valente Alban, Camila Motta Venchiarutti Moniz, Bruno Zilberstein, Evandro Sobroza de Mello, Paulo Marcelo Gehm Hoff, Ulysses Ribeiro, Andre Roncon Dias

**Affiliations:** 1 Universidade de São Paulo, Cancer Institute, Faculty of Medicine, Department of Gastroenterology – São Paulo (SP), Brazil; 2 Universidade de São Paulo, Cancer Institute, Faculty of Medicine, Department of Radiology and Oncology – São Paulo (SP), Brazil; 3 Universidade de São Paulo, Cancer Institute, Faculty of Medicine, Department of Pathology – São Paulo (SP), Brazil.

**Keywords:** Stomach neoplasms, Peritoneal neoplasms, Hyperthermic intraperitoneal chemotherapy, Drug therapy, combination, Catheters, Neoplasias gástricas, Neoplasias peritoneais, Quimioterapia intraperitoneal hipertérmica, Quimioterapia combinada, Cateteres

## Abstract

**BACKGROUND:**

Peritoneal carcinomatosis in gastric cancer is considered a fatal disease, without expectation of definitive cure. As systemic chemotherapy is not sufficient to contain the disease, a multimodal approach associating intraperitoneal chemotherapy with surgery may represent an alternative for these cases.

**AIMS:**

The aim of this study was to investigate the role of intraperitoneal chemotherapy in stage IV gastric cancer patients with peritoneal metastasis.

**METHODS:**

This study is a single institutional single-arm prospective clinical trial phase II (NCT05541146). Patients with the following inclusion criteria undergo implantation of a peritoneal catheter for intraperitoneal chemotherapy: Stage IV gastric adenocarcinoma; age 18–75 years; Peritoneal carcinomatosis with peritoneal cancer index<12; Eastern Cooperative Oncology Group 0/1; good clinical status; and lab exams within normal limits. The study protocol consists of four cycles of intraperitoneal chemotherapy with paclitaxel associated with systemic chemotherapy. After treatment, patients with peritoneal response assessed by staging laparoscopy undergo conversion gastrectomy.

**RESULTS:**

The primary outcome is the rate of complete peritoneal response. Progression-free and overall survivals are other outcomes evaluated. The study started in July 2022, and patients will be screened for inclusion until 30 are enrolled.

**CONCLUSIONS:**

Therapies for advanced gastric cancer patients have been evaluated in clinical trials but without success in patients with peritoneal metastasis. The treatment proposed in this trial can be promising, with easy catheter implantation and ambulatory intraperitoneal chemotherapy regime. Verifying the efficacy and safety of paclitaxel with systemic chemotherapy is an important progress that this study intends to investigate.

## RESUMO

**RACIONAL:**

A carcinomatose peritoneal no câncer gástrico é considerada uma doença fatal, sem expectativa de cura definitiva. Como a quimioterapia sistêmica não é suficiente para conter a doença, uma abordagem multimodal associando a quimioterapia intraperitoneal à cirurgia pode representar uma alternativa para esses casos.

**OBJETIVOS:**

Investigar o papel da quimioterapia intraperitoneal em pacientes com câncer gástrico estágio IV com metástases peritoneais.

**MÉTODOS:**

Trata-se de um ensaio clínico prospectivo unicêntrico, braço único, fase II (NCT05541146). Pacientes com os seguintes critérios de inclusão serão submetidos à implantação de cateter peritoneal para quimioterapia intraperitoneal: adenocarcinoma gástrico estágio IV; idade 18-75 anos; carcinomatose peritoneal com índice de câncer peritoneal<12; *ECOG* 0/1; bom estado clínico e exames laboratoriais dentro da normalidade. O protocolo do estudo consiste em 4 ciclos de quimioterapia intraperitoneal com Paclitaxel associado à quimioterapia sistêmica. Após o tratamento, os pacientes com resposta peritoneal avaliada por laparoscopia serão submetidos à gastrectomia de conversão.

**RESULTADOS:**

O desfecho primário é a taxa de resposta peritoneal completa. A sobrevida livre de progressão e global são outros desfechos avaliados. O estudo foi iniciado em julho de 2022 e os pacientes serão selecionados para inclusão até que 30 sejam inscritos.

**CONCLUSIONS:**

Terapias para pacientes com câncer gástrico avançado foram avaliadas em ensaios clínicos, mas sem sucesso em pacientes com metástase peritoneal. O tratamento proposto neste estudo pode ser promissor, com fácil implantação do cateter e regime de quimioterapia intraperitoneal ambulatorial. Verificar a eficácia e segurança do Paclitaxel associado à quimioterapia sistêmica é um progresso importante que o presente estudo pretende investigar.

## INTRODUCTION

Gastric cancer (GC) is one of the most common cancers, and despite advances in diagnosis and treatment, it remains an important cause of cancer death worldwide^31^. Unfortunately, patients with GC are often diagnosed with clinical stage IV tumors^23,24^.

Among metastatic GC cases, peritoneal dissemination is an important site of progression and tumor recurrence after initial curative intent treatment^26^. It was reported that peritoneal carcinomatosis (PC) is present in 5–20% of patients who are referred for potentially curative surgical treatment^1,3^, and the median survival time without specific treatment is 3–6 months^23,24,28^.

PC is a definitive determinant of prognosis, and the current standard treatment in these cases is systemic chemotherapy (CMT)^14,21^. The CMT for advanced or recurrent GC combines fluoropyrimidine and platinum-based drugs^21^. However, systemic CMT has only a limited effect on peritoneal metastasis, and there is still no established treatment that is effective in treating peritoneal disease^12,17^.

As other therapeutic options, some studies investigating the effect of cytoreductive surgery (CRS) and hyperthermic intraperitoneal chemotherapy (HIPEC) in patients with peritoneal metastases, have suggested an improvement in survival in strictly selected patients^19,27,35,37^. Also, pressurized intraperitoneal aerosol chemotherapy (PIPAC) and normothermic IPC are being studied for patients with PC^2,11,12^.

As intraperitoneal administration of anticancer drugs can induce an extremely high concentration of drugs in the peritoneal cavity, allowing direct contact with peritoneal deposits and free cancer cells in the cavity, IPC seems to be a promising treatment for GC with peritoneal metastasis^12,17^.

To date, taxanes appear to be the most appropriate drugs for intraperitoneal administration to treat peritoneal metastasis of GC – either alone or in combination with systemic chemotherapy^11,17^. These drugs have pharmacokinetic properties that allow high local concentration and rarely cause adhesions in the peritoneal cavity. Studies with taxanes also show that high concentrations of the drug remain for a considerable time, allowing a more prolonged action^11,12^.

Thus, this study was designed to investigate the role of IPC in stage IV GC patients with peritoneal metastasis. The main objective is to evaluate the rate of complete peritoneal response after treatment with normothermic IPC associated with systemic CMT. The current trial is the first in Brazil to evaluate this treatment option for PC, and the results may contribute to a tailored approach to the treatment of patients with GC.

## METHODS

### Hypothesis

We hypothesize that combining IPC with systemic chemotherapy increases the response rate of peritoneal implants with longer survival. In addition, patients with a complete response may be submitted to conversion surgery with still the possibility of disease control and better survival.

### Study design

This is a single institutional one-arm prospective phase II clinical trial, including patients with GC and PC. This study was initiated in July 2022 and is expected to last for 3 years.

The study was approved by the Hospital Ethics Committee (NP 1507/19) and the National Ethics Committee and registered at Plataforma Brasil (CAAE: 26306419.8.0000.0065; https://plataformabrasil.saude.gov.br). The study protocol was registered at ClinicalTrials.gov (NCT05541146).

### Patient Eligibility

All patients with advanced GC referred for staging laparoscopy (SL) for suspected PC will be screened for inclusion. The total sample will be 30 patients. Participants are eligible to be included in the study only if they fulfill the following criteria:

### Inclusion criteria

1. Gastric adenocarcinoma;
2. Age between 18 and 75 years;
3. Eastern Cooperative Oncology Group (ECOG) performance scale 0 or 1;
4. Body mass index (BMI)=18 kg/m²;
5. Presence of PC with intraoperative identification and confirmed by biopsies and/or positive oncotic cytology;
6. Presence of exclusively peritoneal metastasis with peritoneal cancer index (PCI) of ≤12, diagnosed by SL;
7. Patients must have sufficient organ function as follows:
  - Total leukocyte count=3,000/mm^3^;
  - Absolut neutrophil count=1,500/mm^3^;
  - Hemoglobin=8 g/dL;
  - Platelet count=100,000/mm^3^;
  - Total bilirubin <2 mg/dL;
  - Serum alanine transaminase (ALT), aspartate transaminase (AST), alkaline phosphatase (ALP), and gamma-glutamyltransferase (GGT): less than three times the upper reference limit.
    ○ Creatinine clearance=50 mL/min.
8. Expected survival period of ≥6 months.

Exclusion criteria

1. Synchronic or metachronous neoplasms;
2. Previous chemotherapy or radiotherapy for GC;
3. Clinical conditions considered critical for this study by the investigator;
4. Digestive tract obstruction (unless gastric bypass can be performed) and bleeding;
5. Functional class II/III/IV heart failure by the New York Heart Association;
6. HIV infection or chronic use of immunosuppressants;
7. Myocardial infarction and stroke in the past 6 months;
8. Pregnancy.

### Study flow and intervention

**Figure 1:**
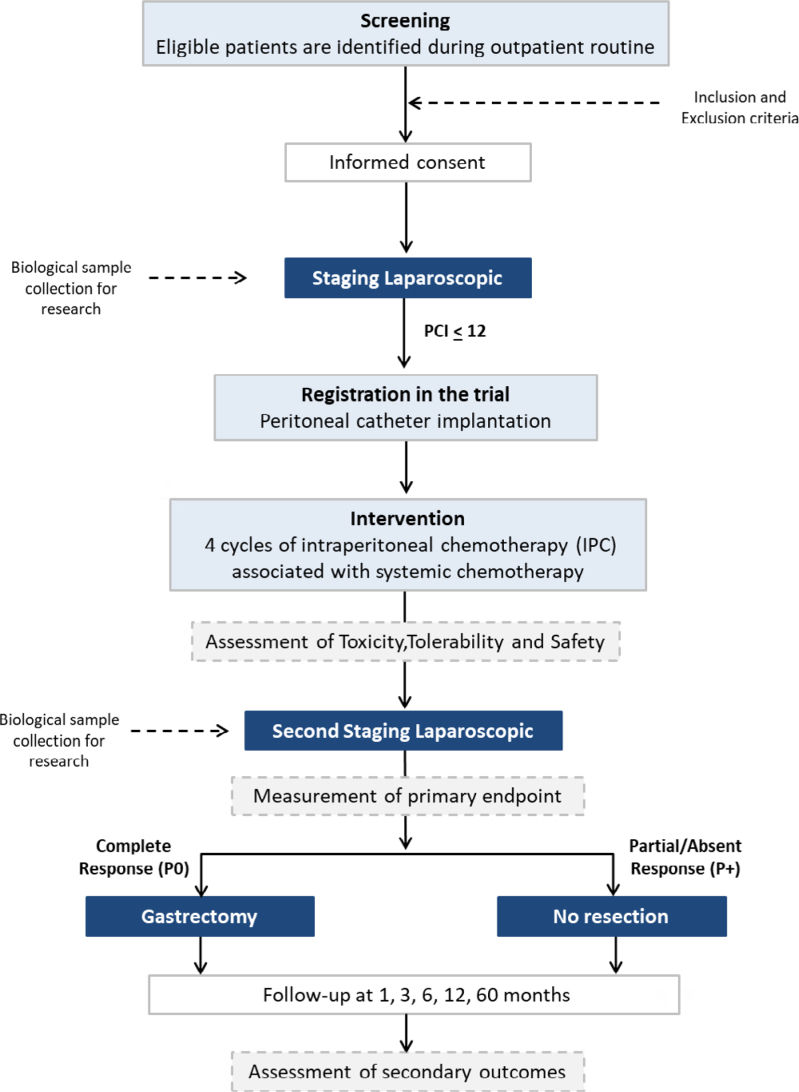
Trial scheme

Newly diagnosed patients with advanced GC will undergo SL and be included in this study when the peritoneal spread is confirmed (with PCI≤12). All patients will undergo standard treatment at the institution with systemic CMT, and the intervention of this protocol will consist of the association of IPC with systemic treatment. The trial scheme is shown in Figure 1.

### Clinical exams and staging

Patients will be evaluated and classified according to the institutional routine, with upper digestive endoscopy, computed tomography (CT) of the chest, abdomen, and pelvis, laboratory tests (blood count, urea, creatinine, sodium, potassium, magnesium, ALT, AST, ALP, GGT, bilirubin, and fractions), and SL.

### Staging laparoscopy and catheter implantation

SL will be performed only by surgeons participating in this study, with extensive experience in the diagnosis and surgical treatment of gastrointestinal cancer. It will be done through an umbilical portal with a 10 mm trocar for optics and two auxiliary portals of 5 mm on the right and left flank.

**Figure 2:**
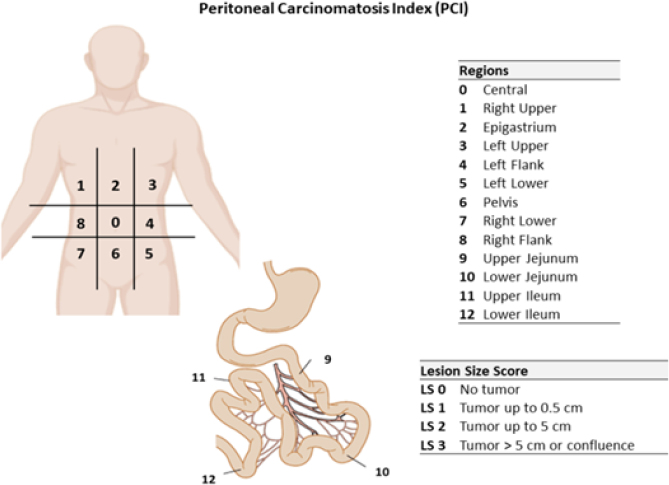
Calculation of the peritoneal cancer index (adapted from Jacquet et al.^13^)

When PC is suspected, a peritoneum biopsy will be performed for the intraoperative frozen section. If the presence of PC is confirmed by the pathologist, the PC index (PCI) will be calculated (Figure 2).

After the confirmation of metastases on the frozen section and a PCI≤12, an intraperitoneal catheter will be inserted. If the PCI is higher than 12, the catheter will not be placed, and the patient will be excluded from the study. In this case, cytoreductive gastrectomy will not be performed, and the patient will be referred for palliative systemic chemotherapy.

A fully implantable long-term catheter (long-term catheter type) will be used (*Celsite*® *Peritoneal Access Port*, T203J – B. Braun Medica, France) and implanted in the subcutaneous tissue of the lower right rib cage, along the middle clavicular line. The catheter will be tunneled for 10 cm in the subcutaneous tissue before penetrating the peritoneal cavity, where another 10 cm of the catheter is placed, with the tip positioned in the pelvic cavity.

After the implantation of the peritoneal catheter, IPC will start after 7 days of implantation up to 90 days. If, for some reason, the treatment is not started within 3 months, the catheter will be removed and the patient will be discontinued from the study.

### Intraperitoneal chemotherapy

The IPC will be performed on an outpatient basis, concomitantly with four cycles of conventional chemotherapy. Paclitaxel (PTX) (40 mg/m^2^) will be infused on D1 and D8, followed by 7 days of rest. The cycle will be repeated every 3 weeks.

The intraperitoneal PTX infusion must be preceded by an intravenous infusion of dexamethasone (20 mg) and diphenhydramine (50 mg), both intravenously, 30–60 min before PTX. Also, the PTX solution will be preceded by an infusion of 500 mL of saline heated in an oven at a temperature of around 37°C (1 h infusion), followed by PTX diluted in 500 mL of saline solution heated to 37°C (1 h infusion). During the infusion, the patient must change positions every 15 min in the following order: right lateral decubitus, left lateral decubitus, supine decubitus, ventral decubitus, and *Trendelenburg.*

Patients will be evaluated before each intraperitoneal infusion, and treatment will be discontinued if inflammatory or infectious peritonitis is suspected, catheter infection is present, or performance deterioration status is noted for ECOG 2. During all the infusions, the presence of infusion-related symptoms will be assessed. Among potential complications, common adverse reactions include mild abdominal discomfort, bloating, diarrhea, and chills. Potentially serious symptoms include any suspected allergic reaction or infection, skin rash, moderate to severe abdominal pain, shortness of breath, hypotension, fever, and chills accompanied by shivering. In these cases, the infusion will be stopped immediately, and the patient will be evaluated by the medical team.

The peritoneal catheter will be removed during the second SL, or earlier if the patient is withdrawn from the study.

### Systemic chemotherapy

All patients must have a pre-therapy CT scan no later than 4 weeks before starting systemic treatment. The chemotherapy used will be preferentially the XP scheme, which consists of oral capecitabine (2,000 mg/m^2^) per day (1,000 mg/m^2^ 12/12 h) from D1 to D14 and intravenous cisplatin (60 mg/m^2^) on D1 every 21 days. The laboratory tests will be performed before D1 and before D8 of each chemotherapy cycle. IPC will be performed concurrently with the first four cycles of XP chemotherapy.

On D1 of each cycle, the infusion of IPC with PXT will be performed before the intravenous cisplatin infusion. On D8, when only PXT is infused, the patient must remain at rest/observation for 2 h after the end of the infusion.

### Study monitoring during CMT

Patients will be closely followed throughout the study and evaluated at each CMT session to monitor adverse events. Complete physical examination, toxicity assessment, and hematological analysis were conducted on D1 and D8 of each cycle. Patients with Grade 3 and Grade 4 toxicity will have their treatment suspended until resolution to Grade 1. CMT will be restarted according to the judgment of the clinical oncologist responsible for the case, and a 20% dose reduction will be recommended, at the investigator's discretion. Follow-up for adverse events will occur for 30 days after the last dose of XP, or until all serious or study-related toxicities are resolved or determined to be chronic or stable.

### Second diagnostic laparoscopy – Restaging after IPC

After the end of the IPC and systemic CMT, patients will be reassessed through clinical and radiological examination, with CT of the chest, abdomen, and pelvis. Those who do not present radiological evidence of disease and maintain ECOG 0 and 1 will undergo a second diagnostic laparoscopy to assess the peritoneal response.

Patients who present a complete response to PC confirmed in LS (negative biopsy of lesions in the peritoneum with negative cytology) and have a primary tumor with potential R0 resection will be submitted to gastrectomy (conversion surgery). After surgery, patients will continue to CMT with XP until cycle 8. Those who present partial response or absence of peritoneal response will have the catheter removed, continuing the standard palliative CMT treatment at the institution.

### Endpoints

The primary endpoint for the current trial will be the peritoneal response rate after four cycles of IPC associated with systemic CMT. The evaluation will be performed during a second SL as follows:

- Complete peritoneal response: the disappearance of all peritoneal lesions, with negative biopsy for tumor cells in the intraoperative frozen section and/or cytology negative
- Partial response: reduction of peritoneal lesions, but with positive cytology and/or positive biopsy for tumor cells in the intraoperative frozen section
- No response: stable disease in the peritoneum
- Disease progression: increase in lesions in the peritoneum

As secondary endpoints, the clinical trial will assess the following parameters:

1. **Toxicity, tolerability, and safety:** Using the Common Terminology Criteria for Adverse Events (CTCAE, v 5.0), these parameters will be evaluated after the treatment.
2. **Postoperative complications (POC):** graded according to the *Clavien-Dindo* classification.
3. **Disease-free survival (DFS):** defined as the time from surgery to metastatic recurrence (only in patients who undergo conversion gastrectomy).
4. **Overall survival (OS):** defined as the time from the start of treatment (IPC D1, Cycle 1) until death due to any cause. Subjects who have not died at the time of the last known follow-up will be censored.
5. **Diagnostic accuracy of peritoneal lavage evaluation methods:** comparison between the conventional cytological technique and liquid-based cytology (LBC) for the detection of tumor cells (sensitivity, specificity, and positive and negative predictive value).

### Material collection and histopathological analysis

#### Sample collection

During the study, samples of peripheral blood, tumor tissue/implant, and peritoneal washing cytology (PWC) will be collected from the patients included in the study. Samples will be collected pre-treatment (at the time of the initial SL) and post-treatment (at the second SL). All samples will be stored in a freezer at a temperature of −80°C, for later study of biomarkers.

#### Peritoneal washing cytology

The peritoneal washing cytology (PWC) will be collected in the operating room. For the procedure, 150 mL of saline solution will be introduced into the abdominal cavity and aspirated after gentle agitation. Three sample tubes (Falcon 15 mL conical tube) with the aspirated material will be collected: one will be destined for conventional cytopathological evaluation (1:1 ratio of 70% alcohol, and processed according to the routine procedure), a second sample will be destined for LBC (added to 10 mL of GynoPrep preservative liquid), and the third will be destined for storage as previously described – which will be centrifuged at 3,000 rpm for 10 min, and the cell pellet will be resuspended with 1 mL of Trizol® (Invitrogen-Life Technologies, Carlsbad, EUA) and stored in a freezer at −80°C. The supernatant will also be stored separately.

Briefly, the sample forwarded to conventional cytology will be centrifuged for 10 min at 1,400 rpm. After the process, the supernatant is discarded and the pellet is resuspended in PBS (pH 7.3–7.4). Thus, the sample is placed in three cyto-funnels (100 μL in each of them) and centrifuged in CytoSpin® for 10 min at 1,400 rpm. After the process, the slides with the smear formed will be removed from the centrifuge and placed in absolute alcohol, for later Papanicolaou staining. In total, each cytological sample will result in three slides for microscopic evaluation. The remaining material from the centrifugation not placed in the cyto-funnel will be packed in histological cassettes (cell block), to be processed in an automated system (Zeiss, STP 420D, Thermo Scientific). Histological sections will be stained with hematoxylin and eosin.

The samples for LBC will be placed in a vortex for 20 s and then processed using the GynoPrep Processor GP-100 (Stra Medical®), a centrifugation system with filters and vacuum. The equipment will perform all the procedures for homogenization, dispersion, and reduction of artifacts and, using a controlled pneumatic mechanism, transfers the cells to be examined to the surface of the slide. After the process, the slides will be fixed in alcohol and also be submitted to Papanicolaou staining and evaluated by conventional microscopy.

The two methodologies will be compared (results and sample quality) to verify which technique presents better performance for the evaluation of the presence of tumor cells in PWC.

### Statistical methods

For this study, the sample size calculation cannot be determined. Based on our experience^24^, we set a sample of 30 patients (convenience sample) for the profile of recruitment established in this study.

Descriptive statistics will include mean (with standard deviation, SD±) and median (interquartile ranges, IQR) for continuous data and absolute and relative frequencies for categorical data. Continuous variables were compared using the standard t-test or Mann-Whitney test, and the chi-square test or Fisher's exact test will be used for categorical variables. Multivariate binary logistic regression, with the odds ratio and the respective 95% confidence intervals, will be used to identify possible factors related to peritoneal response and to identify risk factors related to treatment toxicity.

Survival curves will be evaluated using the Kaplan-Meier method and compared using the log-rank test. The prognostic factors associated with survival will be estimated using the univariate and multivariate Cox proportional hazards model. All statistical tests will be two-sided, and p-values<0.05 are considered significant. Statistical analyses will be performed using the SPSS software, version 20 (SPSS, Chicago, IL).

## RESULTS

The primary outcome is the rate of complete peritoneal response after IPC. Progression-free and overall survivals are other outcomes evaluated. The study started in July 2022, and patients will be screened for inclusion until 30 are enrolled.

## DISCUSSION

The finding of PC in GC is considered uncurable and the final stage of the disease progression. To date, patients with PC only have palliative therapeutic perspectives, presenting an extremely dismal prognosis^17,23,24^. As conventional surgery does not provide a complete tumor resection and systemic chemotherapy treatments are not sufficient to contain the disease, a multimodal approach associated with the use of peritoneal therapies may represent an alternative for these patients^6,14,17^.

In GC with peritoneal metastases, experimental treatment options such as conversion surgery plus HIPEC and normothermic IPC are currently being investigated^2,19,27,36,37^. Although it has demonstrated efficacy for the treatment of peritoneal metastasis in tumors such as ovarian and pseudomyxoma peritonei, HIPEC has a high risk of adhesion. And some studies reported that HIPEC is not considered effective enough to compensate for the morbidity compared to systemic CMT^16,26^.

Although other anticancer drugs for intraperitoneal administration have been used, such as cisplatin and mitomycin C, PTX appears to be a better alternative for IP administration^10,12,17^. PTX is a high-weight lipophilic molecule, which delays its absorption by the lymphatic system, leading to high concentrations for a prolonged time after intraperitoneal administration. Most importantly, the taxanes rarely cause fibrotic adhesions in the abdomen, even when they are repeatedly administered intraperitoneally, in contrast to some other drugs that can cause local chemical peritonitis^10,17^. Thus, surgical resection as a conversion surgery can be technically feasible after IPC.

Despite the advantages, some limitations of IPC are also described. Among them, the depth of infiltration from the surface of the peritoneal disseminated nodules is limited, and the drugs may not reach the deepest part of a large nodule^15,30^. As the effectiveness of IPC may be reduced by the presence of large amounts of peritoneal tumors^35^, we adopted as inclusion criteria patients with a maximum PCI of 12. In a previous trial with GC with PC, all patients surviving beyond 12 months treated with CRS plus HIPEC that achieved a complete macroscopic cytoreduction had an initial PCI of 15^27^.

In fact, the survival benefits obtained with IPC plus systemic CMT for the peritoneal dissemination of ovarian cancer encouraged its use for peritoneal metastasis of GC. Recently, studies on the intraperitoneal administration of CMT for GC have demonstrated encouraging progression^10,11,14,17,19^.

Some studies reported that IPC is efficient in patients with GC who have peritoneal metastasis and can achieve complete regression of lesions in a significant portion of cases^34^. Yamaguchi et al.^35^ conducted a phase II study in 35 patients with GC who had peritoneal metastasis treated with intravenous and intraperitoneal PTX combined with oral S-1. CRS was performed in 21 cases, and the R0 rate was 60%. The 1-year OS rate and median survival rate were 77.1% and 17.6 months, respectively^35^. Similarly, Kitayama et al.^16^ using the same combination of IPC and systemic CMT also showed favorable results for IPC. Among the 64 patients with carcinomatosis included, salvage gastrectomy was performed in 34 (53%) patients who showed significant responses in both peritoneal nodules and cytology. Of these, 22 cases had R0 resections. In patients who undergo gastrectomy, the median survival time and 1-year OS were 26.4 months and 82%, respectively. For those who did not receive gastrectomy, the median survival time was 12.1 months and the 1 year OS was 26%^16^.

Therefore, conversion therapy combining systematic chemotherapy and IPC, followed by surgical intervention, seems to be a better choice for GC patients with PC^11,12,17,19,20^. We have already demonstrated that conversion surgery, including gastrectomy with lymphadenectomy, can have a survival benefit for highly selected patients and the inclusion of peritoneal therapy may increase its indications^22^.

Traditional imaging techniques are not sensitive enough for the detection and evaluation of peritoneal metastasis, particularly due to false-negative rates. SL is the gold standard to confirm the diagnosis and extent of peritoneal metastasis^4,5,6,14,29^. Thus, in this trial, we established that all potential patients will only be included in the study after performing SL, where the PCI will be determined. The PCI is a useful tool to assess disease extensity and can help in determining the prognosis^13^. It is important to highlight that the presence of carcinomatosis is not always visible in SL. So, the evaluation of PWC assumes great importance in the diagnosis of metastases in the peritoneum^25,32^. The usual conventional cytological technique has some limitations, including a high false-negative rate. PWC is positive in only 59% of patients with macroscopic peritoneal disease and is much less sensitive in those without any macroscopic peritoneal metastasis after curative surgery, which is between 5% and 15%^7,18,33^. Also, approximately 10% of patients with negative cytology have a peritoneal recurrence^9^.

To better clarify this issue, this clinical trial will compare the conventional cytological technique with LBC. LBC was developed with the purpose of seeking greater sensitivity for cytological evaluation than the conventional method. By cytology monolayer liquid, it is possible to obtain a monolayer (or thin layer) cytology, as well as a slide with a cleaner bottom, without overlapping cells and obscuring other elements, due to the filter system that causes only epithelial cells to be retained^8^.

## CONCLUSIONS

Finally, it is believed that IPC may not only increase the survival of patients with PC but also offer the possibility of conversion surgery for some patients aiming at a curable intention after tumors are initially deemed oncologically unresectable, as long as IPC shows robust efficacy. So far, the management of peritoneal metastasis has been challenging, and if IPC proves to be effective for some patients, this treatment approach could result in a drastic shift to stage IV GC.
